# Decision aid on breast cancer screening reduces attendance rate: results of a large-scale, randomized, controlled study by the DECIDEO group

**DOI:** 10.18632/oncotarget.7332

**Published:** 2016-02-11

**Authors:** Aurelie Bourmaud, Patricia Soler-Michel, Mathieu Oriol, Véronique Regnier, Fabien Tinquaut, Alice Nourissat, Alain Bremond, Nora Moumjid, Franck Chauvin

**Affiliations:** ^1^ Hygée Centre, Lucien Neuwirth Cancer Institut, CIC-EC Inserm 1408, Saint Priest en Jarez, France; ^2^ EMR3738, Therapeutic Targeting in Oncology, Claude Bernard University, Lyon, France; ^3^ Adémas-69, association pour le dépistage organisé des cancers dans le Rhône, Lyon, France; ^4^ Jean Monnet University, Saint-Etienne, France; ^5^ Lyon 1 University, Lyon, France; ^6^ GATE-LSE UMR 5824 CNRS, Lyon, France; ^7^ Léon Bérard Cancer Centre, Lyon, France

**Keywords:** breast cancer screening, decision aid, informed decision, decision making patient education, randomized control trial

## Abstract

Controversies regarding the benefits of breast cancer screening programs have led to the promotion of new strategies taking into account individual preferences, such as decision aid. The aim of this study was to assess the impact of a decision aid leaflet on the participation of women invited to participate in a national breast cancer screening program. This Randomized, multicentre, controlled trial. Women aged 50 to 74 years, were randomly assigned to receive either a decision aid or the usual invitation letter. Primary outcome was the participation rate 12 months after the invitation. 16 000 women were randomized and 15 844 included in the modified intention-to-treat analysis. The participation rate in the intervention group was 40.25% (3174/7885 women) compared with 42.13% (3353/7959) in the control group (*p* = 0.02). Previous attendance for screening (RR = 6.24; [95%IC: 5.75-6.77]; *p* < 0.0001) and medium household income (RR = 1.05; [95%IC: 1.01-1.09]; *p* = 0.0074) were independently associated with attendance for screening. This large-scale study demonstrates that the decision aid reduced the participation rate. The decision aid activate the decision making process of women toward non-attendance to screening. These results show the importance of promoting informed patient choices, especially when those choices cannot be anticipated.

## INTRODUCTION

Breast cancer is the second most common cancer worldwide and remains the leading cause of cancer death among women [1]. During the last three decades, collective efforts have been made to improve early diagnosis of breast cancer with the aim of decreasing its burden. Breast cancer screening programs have been implemented in numerous countries, mainly as organized population-based breast cancer screening programs [2-6]. The modalities of population-based breast cancer screening vary between countries, but they mainly involved women aged between 50 and 69, with a two-view mammography screening every two years [4, 7, 8]. Although countries aim to achieve 100% participation they actually reach a rate of 50% to 80% [9].

Benefits in terms of mortality reduction are not clearly documented [10-13]. It has been suggested that prevention campaigns should change from persuasive approaches to approaches based on information and women's decision empowerment [14-17].

Decision aids could inform women about the different options available and increase awareness of the benefits and harms associated with breast cancer screening. This would lead women to express their preferences in an informed decision-making process [18, 19]. The development of tools to increase patient empowerment has been strongly endorsed by several organizations in France and elsewhere [20-22].

Although several decision aids are available for breast cancer screening, only a few of them have been properly evaluated and implemented using validated methods [18, 23, 24].

A decision aid, known as the DECIDEO leaflet, was developed following international guidelines for the ‘provision of information and the construction of decision aid tools’ [25, 26]. This tool is a 12-page pocket leaflet presenting the different health decision options and providing probabilities of the outcomes according to the choices made, highlighted by illustrations and histograms [25]. Our hypothesis was that this decision aid would increase informed choice in the intervention group. We estimated the effect of this written decision aid on informed choice, by measuring the participation rate of a population-based breast cancer screening. A randomized, controlled trial in a large sample of French women who were invited to participate in a population-based breast cancer screening program was conducted.

## RESULTS

### Study population

**Figure 1:**
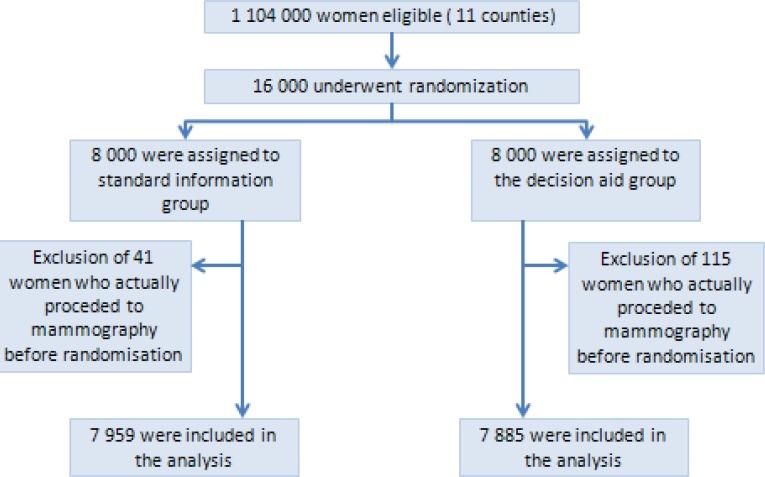
Randomization and follow-up of study participants Some women were excluded because there was a delay between the invitation being sent by the cancer screening association and its reception by the women; during the delay some of the randomized women had already attended breast cancer screening since they did not need to take the invitation letter with them.

Among the 1 104 000 estimated women aged between 50 and 74, registered with the French Health Insurance System of the 11 study departments, 16 000 women were randomized to participate in this study and then randomly assigned to the decision aid group or to the standard information group (in each group *N* = 8 000 Figure 1; e-table 1). A total of 156 women were excluded from the analyses because they had had a mammography within the week before randomization (41 in the control group *vs*. 115 in decision aid group). We collected information about breast cancer screening attendance during the 12 months that followed the expedition of the invitations. The baseline characteristics were comparable between the two groups (Table 1).

**Table 1:** Baseline characteristics of participants (*N* = 15 844) by study group: decision aid (intervention) and standard information (control group)

Characteristics	Decision aid *N*= 7 885 *N* (%)	Standard information *N*= 7 959 *N* (%)	*P* value
Age (years)			
50-59	3 716 (47.1)	3 830 (48.1)	
60-74	4 169 (52.9)	4 129 (51.9)	0.21
Number of invitations already received (leading to participation to national screening or not):			
First	873 (11.1)	824 (10.4)	
One or more	7 005 (88.9)	7 128 (89.6)	0.14
Previous screening attendance			
Yes	3 570 (45.3)	3 746 (47.1)	
No	3428 (43.5)	3462 (43.5)	0.25
Not applicable	887 (11.3)	751 (9.4)	
Household income			
< 25 000 euros/year	4394 (64.4)	4415 (64)	
25 000- 35 000 euros/year	1990 (29.2)	2065 (29.9)	0.48
> 35 000 euros/year	443 (6.5)	423 (6.1)	
Geographical origin			
Urban county	6871 (87.1)	6880 (86.4)	
Rural county	1014 (12.9)	1079 (13.6)	0.20

### Outcomes

A total of 3 174 women in the decision aid group and for 3 353 women in the control group attended breast cancer screening in the 12 months following the invitation. The overall participation rate at 12 months (Table 2), was significantly higher in the control group: 42.13% *versus* 40.25% in the decision aid group (*p* = 0.02).

**Table 2:** Attendance and delay for breast cancer screening by study group [n (%) or median (IQR)]

Outcome	Decision Aid (*N*= 7 885)	Standard information (*N* = 7 959)	*P* value
Attendance at breast screening within 12 months	3 174 (40%)	3 353 (42%)	0.02
Delay to attendance (months)	2.8 (1.3-4.9)	3(1.5-5.1)	0.0025^μ^

μ Wilcoxon test was used since delay was not normally distributed

**Figure 2:**
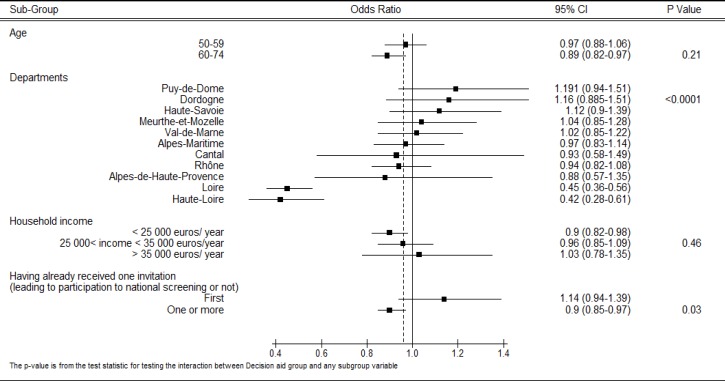
Sub-group analyses to identify baseline characteristics associated with breast cancer screening attendance Results are reported as odds ratios with 95% confidence intervals (CI) (horizontal bar). The dotted vertical line represents the odds ratio in the whole sample (odds ratio 0.86, 95% CI: 0.79-0.94, *P* = 0.0008).

Women in the decision aid group attended the screening earlier (following their invitation) than those in the control group (2.8 months compared with 3 months, respectively, *p* = 0.0025). The cumulative rates of breast cancer screening attendance in each group are shown in eFigure 1. The results from the post-hoc analyses showed that there was a heterogeneous effect of the decision aid (Figure 2). In particular, women living in two departments (Loire and Haute Loire, *p* < 0.0001) or with lower estimated household income (*p* = 0.03) had a lower rate of screening attendance when randomized in the intervention group. The results from the logistic regression analyses showed that being in the intervention group (OR = 0.86; 95%CI [0.79-0.94] *p* = 0.0008), having previously attended breast cancer screening (OR = 15.7; 95%CI [14.2-17.4] *p* < 0.0001) and having an estimated household income of between 25 000 and 35 000 euros (OR = 1.13; 95%CI [1.03-1.2] *p* = 0.01) were independent factors associated with women's attendance for breast cancer screening (Table 3). The results for the Hosmer-Lemeshow goodness of fit test was not significant (*p* = 0.91) and the C-statistic, 0.78, for the area under the curve (AUC) was acceptable.

**Table 3:** Independent factors associated with women's attendance to breast screening

Characteristics	Univariate analysis			Multivariate analysis		
	Odds ratio	95%CI	*p* value	Ajusted Odds Ratio	95%CI	*p* value
Decision aid group	0.91	[0.84 - 0.97]	0.007	0.86	[0.79-0.94]	0.0008
Age in year						
50-59	1	-				
60-74	1.02	[0.95 - 1.1]	0.58			
Number of invitations already received (leading to participation to national screening or not) ^μ^						
First	1	-				
One or More	1.30	[1.02 - 1.66]	0.03			
Previous attendance at breast cancer screening	15.8	[14.2 - 17.4]	<0.0001	15.7	[14.2-17.4]	<0.0001
Household income						
< 25 000 euros/year	1	-		1	-	
25 000 - 35 000 euros/year	1.26	[1.17 - 1.37]	<0.0001	1.13	[1.03-1.2]	0.01
> 35 000 euros/year	1.03	[0.89 - 1.20]	0.67	1.02	[0.85-1.2]	0.86

μ variable not included in the model because correlated to previous attendance at breast cancer screening

## DISCUSSION

This is the fourth study to assess a decision aid for breast cancer screening. Yet it is the first to compare a decision aid for women covered by organised breast cancer screening (aged 50-74), *versus* the usual practice; the first in Europe, and the first with such a large sample [27-29]. Our results demonstrate that a decision aid, designed following specific guidelines, sent with a formal invitation to attend breast cancer screening, resulted in a lower attendance rate and a decrease in the delay of attendance for the women who did participate.

The main result of this study was a decrease in attendance to the national screening in women involved into an informed decision process when compared with the control group. Additionally, we found evidence of geographical heterogeneity for the effect of the intervention, with a major attendance reduction in two departments. Results from our post-hoc analyses suggest that the DECIDEO leaflet discouraged older women, as well as those with a low mean household income, from attending the national breast cancer screening program. It is known that information about potential harm, may dissuade those from lower-level education groups from taking on preventive health behavior, compared with higher-level education groups. The information provided emphasizes the immediacy of the negative consequences and the devaluation of the future benefits of prevention [30]. Additionally, the decision aid was tested with urban women, probably having a high educational level. Women with a lower educational level could have had difficulty in understanding the decision aid. Those two phenomena could partly explain the effect of the DECIDEO leaflet on those specific populations, additional studies being needed to confirm this hypothesis.

To the best of our knowledge, only two randomized trials previously assessed the effect of a decision aid on attendance for breast cancer screening [27, 29]. The Mathieu et al. [27] trial focusing in 70-year-old women found no effect of the decision aid device as regard to the attendance rate. As for Hersch et al [29], they compared two decision aids for screening attendance, with two different internal messages. The scientific question addressed by Hersch was then different to ours: he assessed the effect of information about overdiagnosis on women.

This study is the first to compare a decision aid to real life: the control group consisted of women aged 50 to 74, receiving the usual information about screening. Our results are however in accordance with previous trials in this field suggesting a decreased attendance as results of the implementation of a decision aid on cancer screening [18].

Our study had several limitations. First, we decided not to collect variables assessing decision making process such as knowledge, anxiety, decisional conflict and preferences. However, assessing only the effect of the decision allowed us to eliminate the Hawthorne effect (the women did not know they were being studied). Additionally, the shorter delay for screening attendance for the women in the decision aid group suggests an activation of the decision process by the decision aid, confirmed by the dynamics of screening attendance over time (eFigure 1). Another limitation is that the recent data about overdiagnosis and overtreatment has not been implemented in the decision aid. However, this information is likely to decrease the attendance to screening and might have only amplified the effect of the decision aid in our study. Another limitation of our study is the heterogeneity observed in the subgroup analyses results. Some of this heterogeneity can be explained by the differences in mean annual income, but some variations remain unexplained. Factors, such as competing priorities (budget, employment, children scholarship or other health issues), that may have influenced the decision-making process, were not explored. One last limitation concerns the imbalance in the number of women excluded from the analysis due to screening attendance before reception of the invitation (115 *vs* 41 in the intervention and control groups, respectively).

## CONCLUSIONS

In this large, randomized, clinical trial we observed that the DECIDEO decision aid resulted in decreased breast cancer screening attendance although it accelerated the decision to attend, for those women who did attend. These results suggest that this leaflet have accomplished its main purpose which was to inform the decision-making process.

We believe that our results highlight the dilemma between the goals of population health initiatives and individual choices. However, in the current climate that increasingly favors a balanced, honest presentation of the benefits and harms of screening, it seems important to promote tools for improving informed patient choices.

## MATERIALS AND METHODS

### Trial design

This was a multicenter, controlled trial in which women, who were expecting to receive an invitation for the French national breast cancer screening, were randomized to receive either a decision aid sent with the invitation or the usual standard invitation.

### Complete methods are detailed in e-appendix

### Participants and recruitment

Women, aged between 50 and 74, living in 11 French departments and scheduled to be invited to national screening between May and June 2009 were screened and randomized. Women who had already been diagnosed with cancer, were excluded.

### Randomization

Women aged between 50 and 74 and registered with the French Health Insurance System in the 11 departments were randomly selected through a list-based sample to participate in the study. Women were randomly assigned in a 1:1 ratio *via* computer-generated, centralized randomization sequence, to the DECIDEO or usual invitation group. The randomization was balanced through stratification according to the following hierarchy: the department, the age, and the number of invitations already received by the woman.

The study was approved by an institutional review board (Ethical Committee of Saint Etienne University Hospital (N° IORG004981), December 4^th^ 2008) which waived the need for signed and informed consent according to French law.

### Interventions

#### Decision aid group

Women allocated to the decision aid group received an invitation to participate in the national breast cancer screening program as well as the specially-designed decision aid (a leaflet), by mail. The paper-based leaflet DECIDEO is a 12-page pocket leaflet providing scientific information about the advantages and disadvantages of participating in the national breast screening program, understandable by all, created to conform with the International Patient Decision Aid Standards [25, 26] (a complete decision aid is retrievable in e-appendix).

#### Control group

Women in the control group received an invitation and the usual standard information leaflet, by mail. This invitation is an administrative letter sent to women scheduled to be invited to participate in the national screening program every two years from the age of 50 onwards.

### Outcome measures

Twelve months after the invitations were sent, the participation status of the randomized women was collected, as well as the date of attendance.

#### Primary outcome

The primary outcome was the women's attendance rate for the breast cancer screening program during the 12 months following the invitation.

#### Secondary outcome

The secondary outcome was the delay between the invitation and the date of attendance for breast cancer screening. In addition, the demographic details and other characteristics of the women were collected.

### Sample size

The study sample size was calculated with an assumption of a 50% attendance rate (which was the mean participation rate observed in the 11 participating departments, in 2007). We estimated that there would be a 3% modification in the attendance rate (a 6% relative modification). With an alpha risk of 5% and a beta risk of 95%, we calculated that we needed to include 7 209 women in each group for a bilateral test (since a positive or a deleterious effect of the intervention could equally be possible). To take into account the lost to follow-up and the risk of contamination bias, we increased the group size by 10%, giving a sample size of 8 000 women in each group.

### Statistical analysis

The results were analyzed using a modified intention-to-treat population. The statistical analyses were performed with a Pearson's Chi Square test (Fisher exact test if statistical conditions were not satisfied) for ordinal variables or a Student's T test (Wilcoxon test if statistical conditions were not satisfied) for continuous variables. Variables that were significantly associated with attendance in univariate analyses (*p*. value < 0.05) were introduced in a stepwise manner in a multivariate logistic regression model to identify independent predictive factors (exit *p*.value < 0.05). We also compared the primary outcome in post-hoc defined subgroups to identify potential heterogeneous behavior. All p-values are two sided, with the threshold of significance set at *p* < 0.05. All analyses were carried out using SAS version 9.3.

This study was later registered in clinicaltrial.gov on 03/19/2014, since the principal outcome was a screening attendance rate, therefore not considered as a “health outcome” by the steering committee and the local IRB. Thus, this was considered as off the range, according to the ICMJE definition of a clinical trial at the time of study initiation.

This study was supported by the French National Association against Cancer (Ligue National Contre le Cancer). This organization was not involved, at any time, in the design and conduct of the study.

## SUPPLEMENTARY MATERIAL


