# Supplementary material for: Decision aid on breast cancer screening reduces attendance rate: results of a large-scale, randomized, controlled study by the DECIDEO group
Source: Oncotarget. 2016 Feb 11;7(11):12885–92. doi: 10.18632/oncotarget.7332 (PMC4914328; doi:10.18632/oncotarget.7332)
Supplement: Supplementary file 1 [file oncotarget-07-12885-s001.pdf]

Decision aid on breast cancer screening reduces attendance rate: results of a large-scale, randomized, controlled study by the DECIDEO group

Supplementary Material

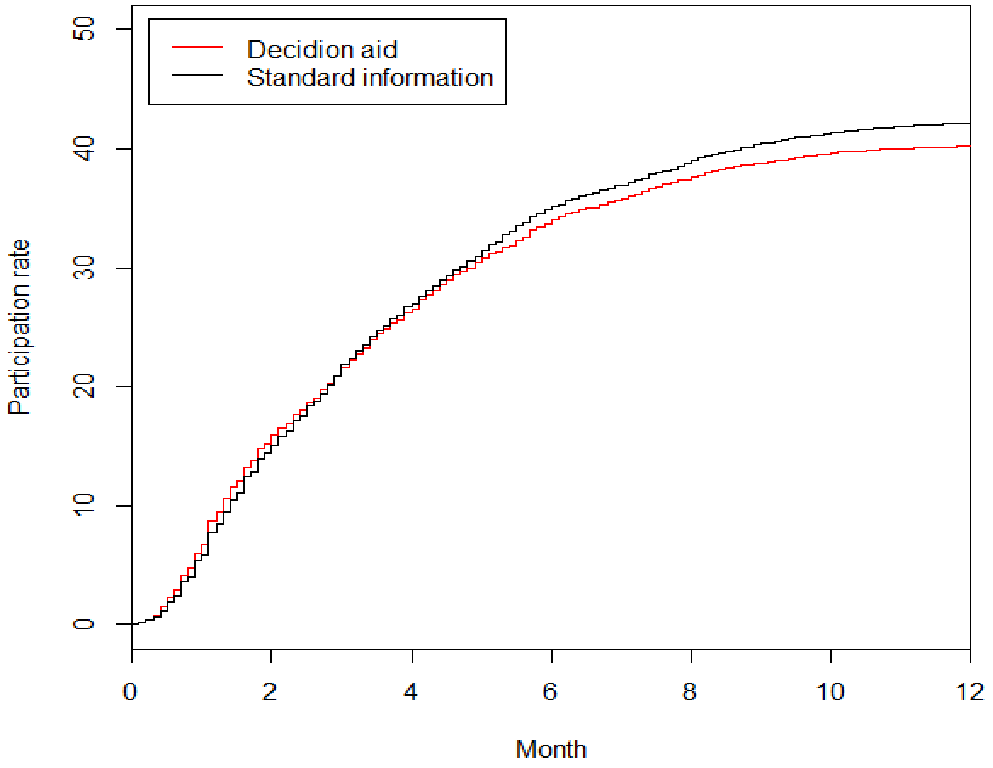

eFigure 1. National breast cancer screening cumulative attendance rates over time, censored at 12 months, for the two intervention groups.

E appendix

1 E results

e-table 1 : number of randomized women by departments, according to the stratification process

departments	randomized women 1:1 ratio			eligible woman ¥ N= 1 104 000	ratio randomized / eligible
	total N= 16000	standard information group N= 8000	decision aid group N=8000		
Alpes-de-Haute-Provence	342	171	171	23 430	1.46%
Alpes-Maritimes	2488	1244	1244	171 487	1.45%
Cantal	371	186	185	24 864	1.49%
Dordogne	986	492	494	68 374	1.44%
Loire	1608	804	804	110 198	1.46%
Haute-Loire	481	240	241	33 155	1.45%
Meurthe-et-Moselle	1464	732	732	102 129	1.43%
Puy-de-Dôme	1372	686	686	96 094	1.43%
Rhône	3168	1584	1584	218 825	1.45%
Haute-Savoie	1366	683	683	93 896	1.45%
Val-de-Marne	2354	1178	1176	161 548	1.46%

¥ source : National Institut of Statistics and Economics Studies (INSEE)

2 E methods

Participants and recruitment

Women, aged between 50 and 74, living in 11 French departments were screened and randomized between May and June 2009. These departments were selected for this study because the participation rate for breast cancer screening was representative of the national participation rate (between 40% and 55%). The women had to be registered with the French Health Insurance System who transmitted their contact details to the cancer screening management structure in their department. Screening management structures (“structures de gestion des dépistages” in French) are devolved administrative organizations that hierarchically depend on the National Health Insurance System. More specifically, they administer the national screening program for breast and colorectal cancer. They manage invitations, tests, and results for all men and women concerned. All cancer screening

management structures in the participating departments took part in the study. Women already diagnosed with cancer were excluded.

Randomisation

Women aged between 50 and 74 and registered with the French Health Insurance System in the 11 departments were randomly selected through a list-based sample to participate in the study. Women were randomly assigned in a 1:1 ratio via a computer-generated, centralized randomization sequence, which was done with a block randomization of four, to the DECIDEO or usual invitation group. The randomization was balanced through stratification according to the following hierarchy: the department, the age according to 2 classes (above or below 65), and the number of invitations already received by the women (leading or not, to participation in national screening)

The study was approved by our institutional review board (Ethical Committee of Saint Etienne University Hospital, December 4th 2008), which waived the need for signed and informed consent according to French law, since the study involved healthy women and did not involve treatment.

Interventions

Decision aid group

Women allocated to the decision aid group received an invitation to participate in the national breast cancer screening program and the specially-designed decision aid (a leaflet), by mail. The paper-based leaflet DECIDEO is a 12-page pocket leaflet providing scientific information about the advantages and disadvantages of participating in the national breast screening program, understandable by all, constructed to conform with the International Patient Decision Aid Standards [33,34] . The aim of DECIDEO was to inform women and to help them reveal their true preferences for breast cancer screening based on scientific evidence. (Supplementary appendix online only). The DECIDEO leaflet was designed by a

multidisciplinary team including physicians, methodologists, sociologists and economists. The leaflet was validated for its psychometric qualities (understandability, reliability, validity) and tested in a sample of targeted women in a monocentre, pilot study (unpublished results).

Control group

Women in the control group also received an invitation and the usual standard information, by mail. This invitation is an administrative letter sent by all the screening management structures to women scheduled to be invited to participate in the national screening program every two years from the age of 50 onwards. The usual standard information document that was sent with the administrative letter was specific to each screening management structure.

Data collection

The French Health Insurance System transmits contact details to the cancer screening management structure in each department. The screening management structures retain individual variables for each woman aged 50 to 74 included in their database. For women included in the DECIDEO study, all screening management structures supplied us with these variables: age, department of living, number of invitations sent, last screening date and postal address. We used those variables to compare baseline characteristics by study groups.

The postal address of each woman was geocoded on global positioning system (GPS) coordinates using Google Map® (Google Inc, California, USA); these coordinates allowed us to attribute 2 sets of variables to women:

- Coordinates allow us to identify for each woman if she was living into a urban or rural area
- Coordinates were linked to the smallest French geographic scale available, the IRIS zone. Each woman were attributed an IRIS zone. The mean household income assigned to the IRIS zone where the woman lived in (source : National Institut of Statistics and Economics Studies (INSEE)) was then attributed to each women.

All those variables were compared by study groups into table 1

Outcome measures

Twelve months after the invitations were sent, each screening management structure collected the participation status of the randomized women. Each structure has the contact details of all women aged between 50 and 74, eligible to receive an invitation to the national screening program for the entire department in a database. This database also records if and when a woman attends screening through automatic transmission of information from the radiologists.

Primary outcome

The primary outcome was the women's attendance rate for the breast cancer screening program during the 12 months following the invitation.

Secondary outcome

The secondary outcome was the delay between the invitation and the date of attendance for breast cancer screening. In addition, demographic details and other characteristics of the women were collected. The level of the household income was estimated based on the mean household income level from the town where the women lived.

Sample size

The study sample size was calculated with an assumption of 50% of attendance rate (which was the mean participation rate observed in the 11 participating departments, in 2007). We estimated that there would be a 3% modification in the attendance rate (a 6% relative modification). With an alpha risk of 5% and a beta risk of 95%, we calculated that we needed to include 7 209 women in each group for a bilateral test (since a positive or a deleterious effect of the intervention could equally be possible). Taking into account the bias of lost to follow-up as well as the risk of contamination , we increased the group size by 10%, giving a sample size of 8 000 women in each group.

Statistical analysis

In compliance with the statistical analysis plan written before study completion, the results were analyzed using a modified intention-to-treat population, defined as all women who were randomly assigned, except those who had had a mammography before randomization. The statistical analyses were performed with a Pearson's Chi Square test (Fisher exact test if statistical conditions were not satisfied) for ordinal variables or a Student's T test (Wilcoxon test if statistical conditions were not satisfied) for continuous variables . Variables that were significantly associated with attendance in univariate analyses (p. value <0.05, conservative as regards to the statistical power of the study) were introduced in a stepwise manner in a multivariate logistic regression model to identify independent predictive factors (exit p.value<0.05). Variables tested positively for correlation were not included in the multivariate analysis. We also compared the primary outcome in post-hoc defined subgroups (Age, Departments, the Household Income and Having or not recieved a previous invitation). Heterogeneity was tested among subgroups. All p-values are two sided, with the threshold of significance set at $p<0.05$. All analyses were carried out using SAS version 9.3.

A steering committee was established to guarantee the accuracy and completeness of the data and the analyses and the respect of the study protocol. The committee validated the decision to submit this manuscript for publication.

2 Decision Aid DECIDEO

English translation is provided on the right of each page

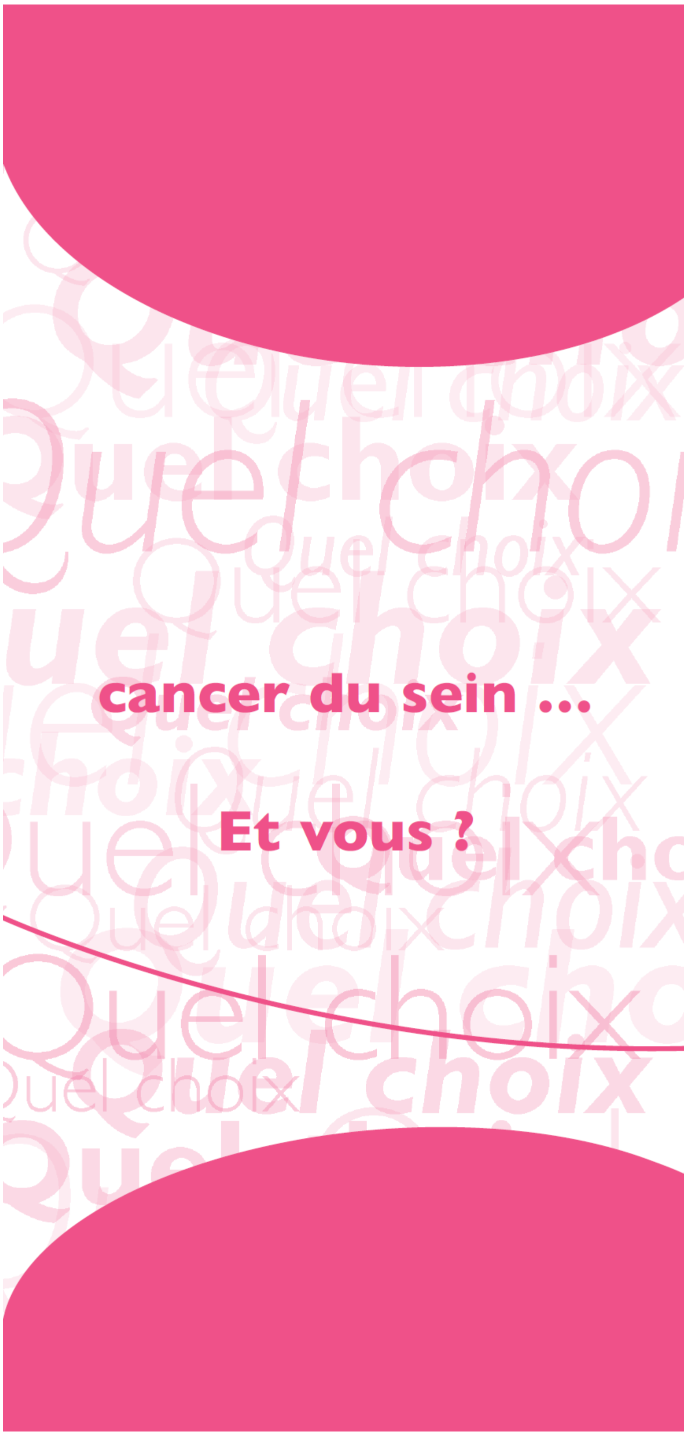

What choices exist?

Breast cancer….What about you ?

Page 1

Connaître les risques du cancer du sein

Le risque que survienne un cancer du sein est lié à l'âge de la femme.

Sur un groupe de 100 femmes que l'on suit de l'âge de 50 ans à l'âge de 80 ans, 10 d'entre elles vont avoir un cancer du sein.

La gravité du cancer du sein est liée à la rechute de la maladie : la récidive locale du cancer au niveau du sein ou l'évolution à distance de la maladie que l'on appelle les métastases. Le risque de décès est lié à cette évolution à distance.

Les risques de rechute et de décès par cancer du sein, dans les 10 ans dépendent de la taille du cancer au moment de sa découverte :

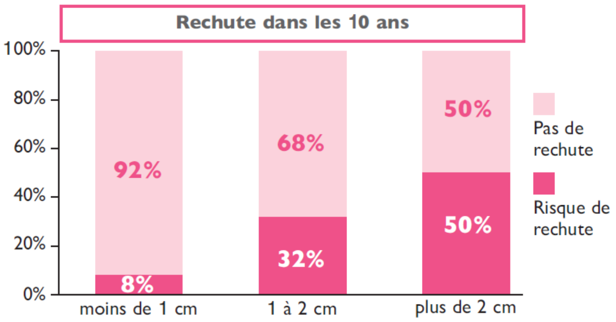

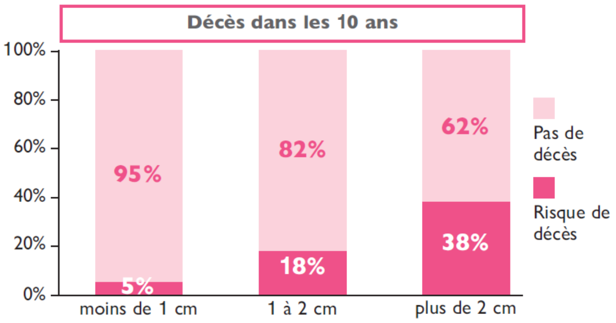

Le cancer du sein se guérit mieux lorsqu'il est de petite taille. Les traitements sont alors moins éprouvants.

Being aware of breast cancer risks

The risk of breast cancer is related to the woman's age.
Among 100 women followed from the age of 50 to the age of 80, 10 will have breast cancer.

The seriousness of breast cancer is related to the relapse of the disease:
The relapse in the localized region of the breast or the evolution of the illness at a distance (also referred to as metastases)
The risk of cancer death is related to this evolution at a distance.

The risks of relapse and death caused by breast cancer in the 10 years following its diagnosis, depend on the size of the tumor at the moment of diagnosis.

Figure 1 : relapse within 10 years
Figure 2 : death within 10 years
Legends :
light pink : no relapse / no death
Fushia : relapse risk / death risk
Abscissa :

- less than 1 cm
- Between 1 and 2 cm
- More than 2 cm

The smaller the breast cancer, the easier it is to cure. Treatment is thus less traumatic.

Connaître le dépistage du cancer du sein

Qu'est-ce que le dépistage du cancer du sein ?

C'est la réalisation répétée d'une mammographie qui consiste en une radiographie des seins. Il permet de trouver le cancer du sein à un stade le plus souvent précoce, avant qu'il ne soit palpable.

- ✓ Une mammographie peut être prescrite par votre médecin : **dépistage individuel.**
- ✓ l'Adémas-69 invite par courrier, systématiquement tous les 2 ans, toutes les femmes de 50 à 74 ans à réaliser une mammographie : **dépistage organisé.**

Les chiffres présentés dans ce document proviennent du dépistage organisé car on ***ne dispose d'aucune donnée chiffrée pour le dépistage individuel.***

Knowing about breast cancer screening

What is breast cancer screening?

It is the repeated realization of a mammography (a breast X-ray). It allows the detection of breast cancer, usually at an early stage, before one can feel it.

- The mammography can be prescribed by your physician : it is **opportunistic screening**
- The national insurance system invite all women between 50 and 74, every 2 years by mail, for a mammography : **population-based national screening**

Figures shown in this document come from the data-base of the national population-based screening since ***no data are available for the opportunistic screening***

Les résultats du dépistage

La mammographie permet de rechercher des anomalies dont certaines seulement sont des cancers.

Sur 1 000 femmes dépistées, 897 ont une mammographie normale (*vrai négatif*).
Sur ces 1 000 femmes, 7 ont un cancer du sein (*vrai positif*).
D'autres anomalies sont découvertes mais elles sont bénignes. Sur les 1 000 femmes, 94 sont dans ce cas (*faux positif*).
Parmi ces 94 femmes, 37 auront besoin d'une surveillance c'est-à-dire d'une échographie et/ou d'une mammographie supplémentaire, plus rapprochées dans le temps.

Pour conclure à l'absence de cancer, il faut parfois faire un prélèvement avec une aiguille (ponction, micro ou macro-biopsie), voire un prélèvement chirurgical.

Deux femmes sur I 000 subiront une chirurgie pour une lésion bénigne.

Breast cancer screening results

A mammography allows the search for anomalies, some of which are cancerous.

For 1000 women screened, 897 have a regular mammography (true negative).
For 1000 women going through screening, 7 are found to have breast cancer (*true positive)*. Other anomalies found are benign. 94 women among the 1000 are concerned (*false positive*).

Among those 94, 37 will need to be followed more frequently with an extra ultrasound and/or a mammography

To confirm the absence of cancer, it is sometimes necessary to make a needle-sample (puncture, biopsy) or a surgical sample.

Two women among 1000 will undergo surgery for a benign anomaly.

Les résultats du dépistage

Certains cancers sont non vus ou non visibles au moment du dépistage. Sur 1 000 femmes dépistées, 1 à 2 sont dans ce cas (*faux négatif*). Elles développeront un cancer dans l'intervalle de temps entre deux mammographies de dépistage. En effet, au moment du dépistage 18% des cancers sont de trop petite taille pour être dépistés. De plus, la densité des seins qui varie d'une femme à l'autre est une cause possible de cancers non vus. Par exemple, un cancer de 1 cm sera bien vu dans un sein « clair » et pourra ne pas être dépisté dans un sein « dense » (cf. photos).

L'organisation mise en place par l'Adémas-69 vise à diminuer le risque de faux négatif.

Parfois la lecture de la mammographie est difficile car les seins sont très denses c'est-à-dire pas assez transparents aux rayons X (cf. photos). Dans ce cas, une échographie peut être nécessaire en plus de la mammographie.

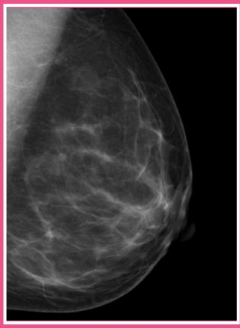

Sein « clair »

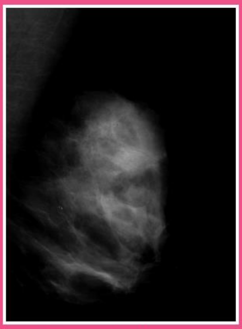

Sein « très dense »

Breast cancer screening results

Some cancers are unseen or cannot be seen through screening. Among 1000 women screened, 1 to 2 will be concerned (*false negatives*). These women will develop cancer between two mammographies. During screening, 18% of the cancer tumors are too small to be diagnosed. Breast density, which varies in women, can be responsible for the non-detection of cancer.
For example a 1 cm cancer tumor could be detected in a "clear" breast and be undetected seen in a "dense" breast. (cf pictures)

The population-based national screening aims at lowering this risk of false negatives

Sometimes it is difficult to read the mammography because breasts are too "dense", i.e. not transparent enough for X rays (cf pictures). In this case, an extra ultrasound can be necessary.

Legends :
"clear" breast
"very dense" breast

Les bénéfices du dépistage

En cas de découverte d'un cancer du sein, les chances de ne pas rechuter dans les 10 ans sont d'autant plus grandes que la tumeur est plus petite. Les femmes qui ont des mammographies régulièrement ont plus de chance qu'on leur découvre un cancer de petite taille.

Le dépistage permet une diminution du risque de décès par cancer du sein :

- Sur 100 femmes qui ne se sont jamais faites dépister et qui ont un cancer du sein traité, 40 décéderont des suites de leur cancer.
- Sur 100 femmes, qui se font dépister tous les deux ans entre 50 et 74 ans et qui ont un cancer du sein traité, 26 décéderont des suites de leur cancer.

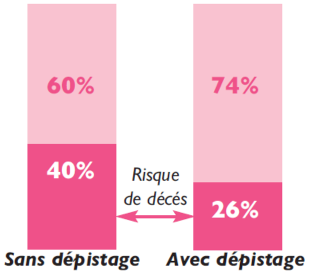

Risque de décès par cancer du sein dans les 10 ans

Le dépistage permet d'avoir des traitements moins éprouvants

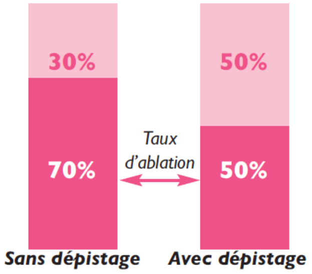

Risque d'ablation du sein

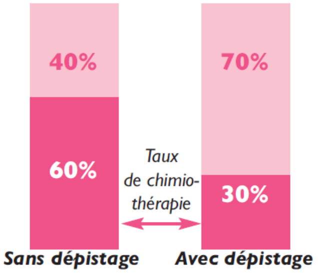

Risque d'avoir une chimiothérapie

Le traitement par radiothérapie est le même dans les deux cas

Vous pouvez comparer les avantages

Screening advantages

In the case where breast cancer is discovered, the smaller the tumor is, the lower the chances are for relapse within the 10 following years. Women who have regular mammographies have more chance of being diagnosed with a small tumor.

Screening allows a decrease in death caused by breast cancer.

- Among 100 women who have never undergone screening and who have been diagnosed with and treated for breast cancer, 40 will die from this cancer
- Among 100 women screened every two years between 50 and 74 years old, diagnosed with and treated for breast cancer, 26 will die of this cancer

Figures Legends :

Death risk

Without screening ***With screening***

Breast cancer death risk within 10 years

Screening allows less traumatizing treatment

Ablation rate

Without screening ***With screening***

Breast ablation risk

Chemotherapy rate

Without screening ***With screening***

chemotherapy risk

radiotherapy treatment is equally frequent in both cases

You can compare advantages

Les inconvénients du dépistage

- **Une douleur modérée : chez 700 femmes sur 1000, la douleur est cotée en moyenne 3 sur 10.** Elle est due à la compression indispensable des seins au moment de la mammographie.
- **Des risques dus aux rayons X** reçus lors d'une mammographie. Ils sont minimes : 1 décès par cancers induits pour 100 000 femmes régulièrement dépistées, mais dans le même temps 50 décès par cancer du sein évités.
- **Une image anormale* peut entraîner :**
  - ✓ **des examens complémentaires :** une échographie, de nouveaux clichés, ou une éventuelle biopsie sous anesthésie locale.
  - ✓ **une inquiétude** avant que tous les examens soient réalisés.
  - ✓ **des consultations et des mammographies** plus fréquentes.
  - ✓ **une opération chirurgicale** sous anesthésie générale pour une anomalie qui s'avère bénigne.

* **Attention** une image anormale ne veut pas dire qu'il y a un cancer.

- **Cancer non vu ou non visible :**

Pour diminuer le risque de cancer non vu ou non visible, il faut :
- ✓ s'examiner les seins chaque mois
- ✓ consulter un médecin à la moindre anomalie

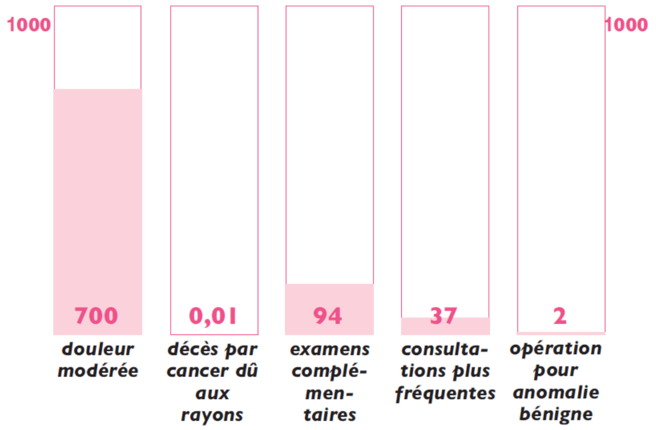

Pour 1000 femmes participant au dépistage

et les inconvénients du dépistage

Page 7

Screening disadvantages

- **Mild pain : for 700 women among 1000, pain is assessed at 3 out of 10 on the pain scale, on average.** It is due to the necessary compression of the breast for the mammography
- **X ray risk** received during mammography. They are minimal : 1 radio-induced cancer death for 100 000 women screened, versus 50 breast cancer deaths avoided
- **An abnormal image*may lead to :**
  - **Complementary examinations :** ultrasound, further X rays, or possibly a biopsy with a local anesthetic
  - **Anxiety** before all the examinations are over
  - **More frequent consultations and mammographies**
  - **Having surgery** with a general anesthetic, for an eventually benign anomaly

***Beware,** an abnormal image doesn't necessarily indicate cancer

- **unseen or invisible cancer :**

To lower this risk, it is important to :
  - practice self-examination every month
  - consult a physician for any anomaly

Legends :
Mild pain
Radio-induced cancer death
Complementary examinations
More frequent consultations
Surgery for benign anomaly

For 1000 screened women

Screening disadvantages

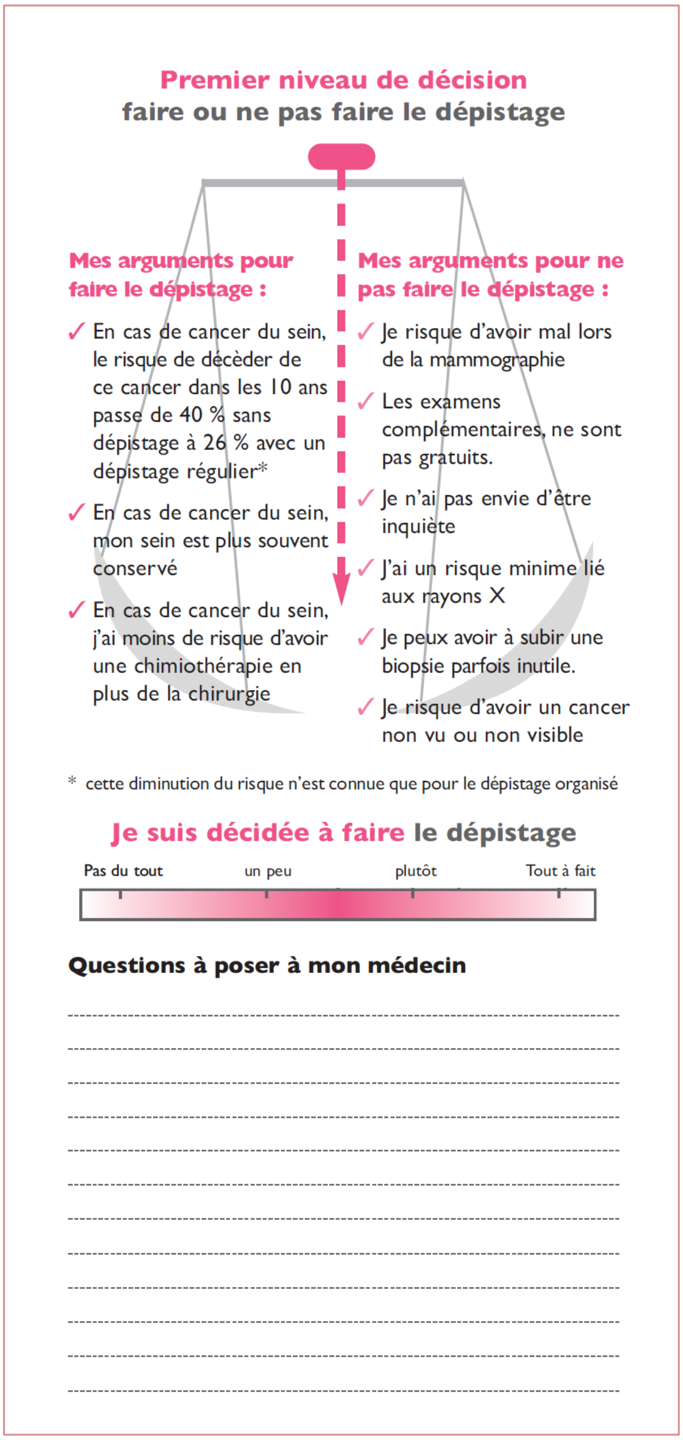

Premier niveau de décision
faire ou ne pas faire le dépistage

Mes arguments pour faire le dépistage :

- ✓ En cas de cancer du sein, le risque de décèder de ce cancer dans les 10 ans passe de 40 % sans dépistage à 26 % avec un dépistage régulier*
- ✓ En cas de cancer du sein, mon sein est plus souvent conservé
- ✓ En cas de cancer du sein, j'ai moins de risque d'avoir une chimiothérapie en plus de la chirurgie

Mes arguments pour ne pas faire le dépistage :

- ✓ Je risque d'avoir mal lors de la mammographie
- ✓ Les examens complémentaires, ne sont pas gratuits.
- ✓ Je n'ai pas envie d'être inquiète
- ✓ J'ai un risque minime lié aux rayons X
- ✓ Je peux avoir à subir une biopsie parfois inutile.
- ✓ Je risque d'avoir un cancer non vu ou non visible

* cette diminution du risque n'est connue que pour le dépistage organisé

Je suis décidée à faire le dépistage

Pas du tout — un peu — plutôt — Tout à fait

Questions à poser à mon médecin

First step of decision-making
Undergoing or not undergoing screening

My arguments in favor of screening :

- In case of breast cancer, death-associated risk in the next 10 years moves from 40% without screening to 26% with regular screening*
- In case of breast cancer, my breast is more often preserved
- In case of breast cancer, I have less risk of having chemotherapy with the surgery

My arguments against screening :

- I may experience pain during mammography
- Complementary examinations are not free of charge
- I don't want to worry about this
- I have a little risk related to X ray
- I could be subjected to a biopsy , that may be unnecessary
- I still risk having an unseen or invisible cancer

*this reduction rate is available only for population based national screening

I am determined to attend screening:

Legends :
Not at all, a little, rather yes, absolutely

Questions I want to ask to my physician:

….

Dépistage organisé ou individuel ?

Rappel

Dépistage individuel :
le médecin prescrit une mammographie sans passer par l'Adémas-69.

Dépistage organisé :
l'Adémas-69 envoie, tous les 2 ans, à toutes les femmes de 50 à 74 ans une lettre d'invitation à pratiquer une mammographie.

Dépistage organisé (Adémas-69)	*Dépistage individuel*
Rappel régulier et automatique de la date de l'examen	Rappel par le médecin lors d'une visite
Contrôle de la qualité technique des clichés	
Double lecture systématique à l'Adémas-69 des mammographies qui ne sont pas suspectes (cf. verso)<br>6 cancers sur 100 découvert par la seconde lecture	Pas de double lecture systématique<br>Pas de données chiffrées
Résultat immédiat et double lecture des clichés remis 15 jours plus tard	Résultat immédiat et pas de double lecture
Relecture possible de la mammographie par le médecin traitant ou le gynécologue	
Pas d'avance des frais pour la mammographie : gratuite	Avance des frais de la mammographie
Avance des frais des examens complémentaires	

Breast cancer screening
Population-based or opportunistic?

- **Reminder** :

Opportunistic screening:
The physician prescribes a mammography without the intervention of the Health insurance system

Population-based national screening :
The Health Insurance system mails invitations every 2 years to women aged between 50 and 74 to undergo a mammography.

**Population-based national screening**	**Opportunistic screening**
Regular and automatic reminder of the examination	Reminded by the physician during a consultation
Quality control of the X rays	
Double reading of the unsuspected X rays	No double reading
6 cancers diagnosed thanks to double reading	No data
Immediate results plus double reading results in 2 weeks	Immediate results , no double reading
Extra reading by the physician always possible	
No need to advance fees : free mammography	Need to advance fees for the mammography
Need to advance fees for every complementary examination	

La double lecture spécifique au dépistage organisé, consiste en une seconde lecture à l'Adémas-69 par un autre radiologue spécialement formé. En effet, si le radiologue qui a réalisé votre mammographie ne voit pas d'image anormale, les clichés sont envoyés à l'Adémas-69 et relus par un autre radiologue spécialement formé à cette double lecture. Les résultats de cette double lecture sont remis 15 jours plus tard.

- ✓ Si une anomalie est vue lors de cette double lecture, vous en êtes informée par courrier recommandé.
- ✓ Si la qualité technique des clichés est insuffisante, l'Adémas-69 demande de les refaire. Cela concerne 0,3% des mammographies.
- ✓ L'Adémas-69 est informée et suit la réalisation du contrôle qualité des appareils.
- ✓ Dans tous les cas, votre médecin traitant ou votre gynécologue peuvent continuer à vous suivre, les résultats du dépistage organisé leur sont communiqués.

Pour 100 cancers du sein concernant des femmes se faisant dépister

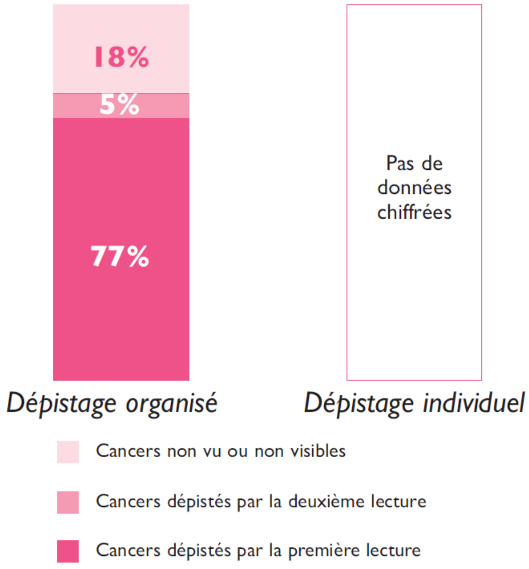

The X-ray double reading is specific to national population-based screening : it is an extra X- ray reading performed by a specially-trained independent radiologist . If the radiologist who performed the X-ray doesn't see any anomaly, X-rays are sent to a structure and read a second time by a specially trained radiologist. Results are sent 15 days later.

- If an anomaly is detected during this double reading, you are informed by mail
- If the quality of the X-ray is insufficient, you are asked to perform another mammography. 0.3% of all mammographies are concerned
- The national health insurance system and its structures are informed and follow the quality control of all X-ray machines.
- In all cases, follow-up by your general practitioner or gynecologist is possible. Screening results are sent to them as well.

For 100 breast cancers related to screened women:

Legends :
No data
National population-based screening/ Opportunistic screening

Very light pink : unseen or invisible cancers
Light pink : double reading diagnosed cancers
Fushia : first reading diagnosed cancers

Vous avez le choix entre ces deux formes de dépistage

Second niveau de décision :
faire le dépistage individuel ou participer au dépistage organisé.

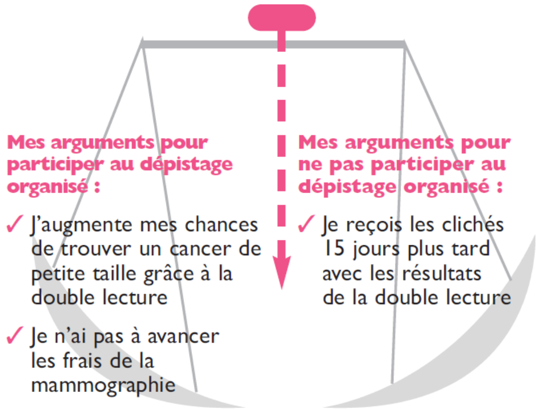

Je suis décidée à participer au dépistage organisé

Pas du tout — un peu — plutôt — tout à fait

Questions à poser à mon médecin

You have the choice between these 2 types of screening

Second step in decision-making
Attending opportunistic or National population-based screening

My arguments in favor of population-based screening :

- I have more chance to be diagnosed with a small sized cancerous tumor, thanks to the double reading
- I do not need to advance fees

My arguments against population-based screening :

- I receive results 15 days later, because of the double reading

I am determined to attend population-based screening:

Legends :
Not at all, a little, rather yes, absolutely

Questions I want to ask to my physician:
….

Références bibliographiques

Données générales : incidence, mortalité, bénéfices et effets secondaires.

- American Cancer Society : cancer facts and figures 2003. Atlanta, Ga : American Cancer Society, 2003.
- IARC Handbook of cancer prevention. Volume 7. Breast cancer screening. Lyon. IARC Press. 2002
- PDQ Screening for Breast Cancer. **www.cancer.gov/cancerinfo/pdq/screening/breast**
- Sharp P.C. et al. Reported pain following mammography screening. Archives of Internal medecine. 2003 ; 163 : 833-836.
- Institut national du cancer : Dépistage organisé du cancer du sein : rapport InVs – InCa : **www.e-cancer.fr/v1/fichiers/public/point_depistage_sein_invs_inca_201006.pdf**
- Institut de veille sanitaire : **www.invs.sante.fr/publications/2008/depistage-cancer-sein/depistage-cancer-sein.pdf**

Bénéfices du dépistage

- Fielder HM et al. A case-control study to estimate the impact on breast cancer death of the breast screening program in Wales. Journal of medical screening. 2005 ; 12 : 107.
- Miltenburg GA et al. Seventeeth-year evaluation of breast cancer screening: the DOM project, The netherlands. British journal of cancer. 1998 ; 78 : 962-965.
- Samnakay N. et al. Rates for mastectomy are lower in women attending a breast-screening programme. Australian and New Zealand journal of surgery. 2005 ; 75 : 936-9.
- Freedman GM et al. Routine mammography is associated with earlier stage disease and greater eligibility for breast conservation in breast carcinoma patients age 40 years and older. Cancer. 2003 ; 98 : 918-25.
- Ernst MF et al. Breast carcinoma diagnosis, treatment, and prognosis before and after the introduction of mass mammographic screening. Cancer. 2004 ; 100 : 1337-44.
- Carter et al. Relation of tumor size, lymph node status and survival in 24 740 breast cancer cases. Cancer 1989 ; 63 : 181-187

Pemaco 04 78 62 73 77

Pour plus de renseignements :
www.ademas69.asso.fr

ou écrire à l'adresse ci-dessous
Adémas-69
5, bis rue Cléberg 69322 Lyon Cedex 05
Tél : 04 72 84 65 30
Fax : 04 72 84 65 39
contact@ademas69.asso.fr

adémas69
association pour le dépistage organisé des cancers dans le Rhône

References:

General data: incidence, death rate, advantages, side effects

Population based screening advantages

We included illustrations in the leaflet, since it has been demonstrated that combining illustrations with text enhances attention, recall and understanding (29). The leaflet provided information on breast cancer and the risks associated with the outcome of the different diseases. It also presented the different screening options and provided the risks associated with the outcome, according to screening choices (with or without). It then went on to compare the risk associated with the outcome between participation in population-based or individual screening. The risks were presented using color-coded histograms. To illustrate how the women should apply the information in order to make their decision about screening attendance, we used the image of weighing scales to represent the decisional steps. One of the scales represented the choice between screening or no screening: They summarized the advantages of breast screening, (e.g. better prognosis, quality of life) on the one hand, and the disadvantages (pain, failure to diagnose a cancer) on the other. The scales were drawn in balance, to allow the women to make their own assessment and choice. The second scales illustrated the choice between population-based screening and individual screening. They summarized the characteristics and advantages of population-based screening (e.g. double centralized reading of the mammogram, third-party payer) and its advantages over individual screening (which has disadvantages too, for example, you cannot take your mammography home). The scales were also drawn in balance in this second decisional step. At the end of the leaflet there was a blank page with the following words: ‘questions to ask my doctor:’
